# Therapeutic alliance and predictive validity of reflective functioning in conjoint mentalization-based treatment for borderline personality disorder

**DOI:** 10.3389/fpsyg.2026.1919516

**Published:** 2026-10-09

**Authors:** Dag Anders Ulvestad, Elfrida Hartveit Kvarstein, Andreas Ekberg, Peder Ludvig Sætre, Geir Pedersen, Theresa Wilberg

**Affiliations:** 1 Clinic for Group Therapies and Personality Disorders, Division of Mental Health and Addiction, Nydalen District Psychiatric Center, Oslo University Hospital, Oslo, Norway; 2 Institute of Clinical Medicine, University of Oslo, Oslo, Norway; 3 Division of Mental Health and Addiction, Department of Research and Innovation, Oslo University Hospital, Oslo, Norway; 4 Quality Laboratory of Psychotherapy, National Advisory Center for Substance Use and Addictive Disorders, Severe Concurrent Mental Health Disorders and Personality Disorders, Oslo University Hospital, Oslo, Norway

**Keywords:** borderline personality disorder, group psychotherapy, individual psychotherapy, mentalization-based treatment, predictor, reflective functioning, therapeutic alliance

## Abstract

**Objective:**

Few studies have investigated the therapeutic alliance in treatment of patients with borderline personality disorder (BPD), particularly regarding alliance in group psychotherapy. This study aimed to investigate the development of therapeutic alliance in a 3-year outpatient mentalization-based treatment (MBT) program for BPD combining individual and group psychotherapy, and to examine whether reflective functioning (RF) predicted alliance development.

**Method:**

Patients with BPD or significant sub-threshold borderline personality pathology were included, with baseline data from 47 patients and longitudinal data from 42. Baseline assessment comprised diagnostic interviews, self-report measures, and interview-based measures of RF. Patient-reported alliance was assessed every 6 months using the Working Alliance Inventory (subscales Bond, Task and Goal) for individual psychotherapy and the Group Questionnaire (domains Positive Bond, Positive Work and Negative Relationship with their respective subscales) for group psychotherapy. Main statistical method was linear mixed models.

**Results:**

In individual psychotherapy, initial alliance levels were satisfactory across all subscales. Over the course of treatment, the Bond level remained stable, whereas Task and Goal showed small but statistically significant increases. In group psychotherapy, Positive Bond was high initially and stable during treatment. For the Positive Work domain and its two subscales, initial levels were below the satisfactory range. During treatment, there was a small but significant increase in agreement on tasks and goals with the other group members but not with the therapists. For the Negative Relationship domain and its three subscales, all estimates except one were within the satisfactory range initially. Initial level of the subscale Group Members was outside the satisfactory range, indicating somewhat strained relationships with the other group members. Longitudinally, there were no improvements for the domain or subscales. RF at baseline had no significant effect on the therapeutic alliance in either treatment modality.

**Conclusion:**

The study demonstrates overall satisfactory levels of therapeutic alliance in an MBT program for BPD but also indicates specific alliance-related challenges in the group psychotherapy component. Initial level of RF may not affect the therapeutic alliance in MBT treatment.

**Clinical trial registration (if applicable):**

ClinicalTrials.gov ID NCT04157907.

## Introduction

Therapeutic alliance is a pan-theoretical concept applied across different treatment approaches and modalities, and one of the most investigated factors influencing treatment outcome (Fluckiger et al., 2018; Lo Coco et al., 2022; Henderson et al., 2026). In the treatment of borderline personality disorder (BPD), establishing and maintaining a therapeutic alliance can be particularly challenging, thus potentially undermining treatment compliance and outcome (Notsu et al., 2024; Levy et al., 2010). Yet, empirical research on alliance in the treatment of BPD remains limited, especially regarding how it develops over the course of therapy (Folmo et al., 2021; Kratzer et al., 2024). The present study examines the levels and course of the therapeutic alliance and the predictive validity of reflective functioning in conjoint individual and group mentalization-based treatment (MBT) for BPD.

In individual psychotherapy, the alliance has been conceptualized in various ways, such as therapeutic alliance, helping alliance, or working alliance (Zetzel, 1956; Luborsky, 1976; Bordin, 1979). In Bordin's (1979) pan-theoretical model—widely influential in psychotherapy research—the working alliance comprises the affective bond between patient and therapist and their agreement on tasks and goals of treatment, thereby emphasizing the collaborative stance in the therapeutic dyad. In the present study, therapeutic alliance denotes alliance in both individual and group psychotherapy. In group psychotherapy, however, the conceptualization of the therapeutic alliance is more complex. Two dominant perspectives define it in terms of either the group's structural properties or the quality of the group relationships (Krogel et al., 2013). The group relationship structure is tripartite, i.e., the relationships of group member to therapist(s), group member to the other members, and group member to the group as a whole (Burlingame et al., 2002). The qualitative aspects usually comprise group cohesion, group climate, alliance, and empathy, assessed by a range of different measures (Henderson et al., 2026; Krogel et al., 2013; Burlingame et al., 2018). The Group Questionnaire (GQ) is a self-report measure which assesses these variables as well as agreement on tasks and goals, across the tripartite group relationship structure, thereby integrating the two dominant views on therapeutic alliance in group psychotherapy (Krogel et al., 2013).

The alliance in individual psychotherapy has been extensively investigated, with strong evidence for its robust positive association with treatment outcome, with small-to-medium effect sizes (Fluckiger et al., 2018; Horvath et al., 2011). Alliance in group psychotherapy has received less research attention, and findings regarding its association with clinical outcomes have been mixed. Nonetheless, it appears to be a contributing factor to patient improvement and lower dropout (Lo Coco et al., 2022; Henderson et al., 2026; Burlingame et al., 2018; Alldredge et al., 2021; Stoffers-Winterling et al., 2022). For patients with BPD, however, research on the therapeutic alliance remains scarce, irrespective of treatment modality (Folmo et al., 2021; Kratzer et al., 2024; Euler et al., 2018). BPD is characterized by a pattern of instability in interpersonal relationships, self-image, and affects, and marked impulsivity (APA, 2013). It is a highly prevalent and debilitating condition associated with poor quality of life, impaired psychosocial functioning, and high societal costs of illness (Ellison et al., 2018; Wibbelink et al., 2025; Sveen et al., 2024). Over the last decades, there has been growing treatment optimism regarding BPD, and there is reasonable evidence that psychotherapeutic interventions, including MBT, are helpful for this condition (Stoffers-Winterling et al., 2022; Yin et al., 2026; Storebo et al., 2020).

Yet, several characteristic features of BPD may impact the development and stability of the therapeutic alliance, such as insecure or disorganized attachment, epistemic mistrust (i.e., an impaired capacity to recognize others as reliable and relevant sources of information and to remain open to knowledge transmission), unstable emotional and cognitive states, high sensitivity to perceived misunderstanding or rejection, proneness to regression and acting out, and alternating idealization and devaluation of the therapist or fellow patients (Bender, 2005; Skodol et al., 2002; Bröcker et al., 2026; Nolte et al., 2023). Therapeutic alliance is emphasized as a crucial but vulnerable factor in the treatment of BPD, but only a few studies report on the longitudinal development of alliance (Folmo et al., 2021; Kratzer et al., 2024). In a study of skills use and treatment processes in dialectical behavior therapy (DBT), the therapeutic alliance was assessed by self-report every 2 months over 1 year (*N* = 70), with alliance levels found to be satisfactory early in treatment and stable across the study period (Barnicot et al., 2016). In another study (*N* = 101), the alliance in DBT was compared with community treatment by experts (Bedics et al., 2015). During the 1-year treatment, patient-rated alliance was assessed after the first session and at 4-month intervals until termination. In both treatments, alliance levels were satisfactory initially and increased toward the end of treatment.

Group psychotherapy is a common treatment format for BPD, yet research on the development of therapeutic alliance in this context is limited. In a 30-session randomized, controlled trial for BPD, patient-rated therapeutic alliance was assessed early (session 3) and later (session 8) in, respectively, interpersonal group psychotherapy (*n* = 16) and individual dynamic psychotherapy (*n* = 18; Marziali et al., 1999). In both modalities, the alliance was rated as moderately positive at both time points, with no statistically significant differences between early and later ratings. Moreover, in a study of a 20-week DBT skills group for patients with suicidal behaviors and BPD (*N* = 42), alliance was assessed repeatedly during treatment (Stratton et al., 2020). Initial alliance was considered below satisfactory levels (Duncan et al., 2003), but due to missing data, it was not possible to draw valid conclusions about the longitudinal development.

With regard to MBT for BPD, only one study has investigated the development of working alliance during treatment (Folmo et al., 2021). It was conducted during an earlier period at the same clinic as the present study, focusing solely on the individual therapy. Initial levels of alliance were satisfactory, and good-outcome therapies were characterized by a process in which the alliance strengthened over time. However, MBT is typically provided as a combined individual and group treatment, with the latter regarded as a core treatment component (Barrett et al., 2025; Karterud and Bateman, 2010). To our knowledge, no study has examined the development of the therapeutic alliance in outpatient MBT programs for BPD, focusing on both individual and group therapy.

Despite alliance challenges in BPD treatment, few studies have investigated patient characteristics that may affect it. Studies investigating the significance of current symptom distress have shown inconsistent results. In the study of Marziali et al. (1999), neither pretreatment symptom distress nor social functioning affected the alliance, whereas more pretreatment problem behaviors predicted poorer patient-rated alliance in the group therapy. In the study of DBT and supportive therapy by Richardson-Vejlgaard et al. (2013), however, higher baseline patient-reported depression, anxiety, and hopelessness predicted lower patient-rated alliance after 2 months of treatment. A few other studies have examined personality or variables related to personality functioning. High agreeableness predicted stronger session-by-session working alliance in short-term Good Psychiatric Management for BPD (Kramer et al., 2023). Poorer executive attention was associated with lower working alliance across three different treatments for BPD, but this relationship was mediated by vacillations in sense of self and others (Levy et al., 2010). In a cross-sectional study of inpatient MBT group therapy, interpersonal sensitivity, in terms of threat of potential exclusion, was found to predict alliance impairments (Euler et al., 2018).

One possible patient-related predictor of therapeutic alliance is mentalizing capacity, a social-cognitive capacity operationalized as reflective functioning (RF), referring to the ability to understand one's own and others' behavior in terms of underlying mental states (Fonagy and Luyten, 2009). A growing body of research indicates that impaired RF characterizes many forms of psychopathology, but is a core feature of BPD, where patients typically show low and fluctuating RF (Luyten et al., 2020). Research suggests that RF may moderate psychotherapy outcome and might be a mediator of change (Luyten et al., 2024). RF has also been theorized to foster epistemic trust, thereby potentially enhancing treatment engagement and alliance in psychotherapeutic settings (Bröcker et al., 2026; Nolte et al., 2023; Antonsen et al., 2016; Bouchard et al., 2008; Jørgensen et al., 2021). Against this theoretical and empirical backdrop, it has been assumed that the stimulation of the mentalizing capacity is a common mechanism of effective psychotherapies, given that psychotherapeutic processes across modalities invariably involve some degree of mentalizing (Jain and Fonagy, 2020; Fonagy and Allison, 2014). A recent systematic review, however, has provided a more differentiated picture by demonstrating that psychoanalytic and psychodynamic therapies produce greater improvements in RF than CBT (Luyten et al., 2024). In MBT, which is rooted in contemporary psychodynamic theory, psychoanalysis, and attachment theory, RF is an explicit treatment target, and for patients with BPD an important treatment outcome in its own right (Leichsenring et al., 2024; Fonagy et al., 2015). The typically low levels of pretreatment RF may undermine emotional regulation, interpersonal trust, and the collaborative engagement necessary to establish and sustain a satisfactory alliance (De Salve et al., 2025; Kivity et al., 2021). Although RF can improve over the course of therapy, for many patients these changes may be modest, and impaired RF thus may remain a clinically relevant vulnerability (Katznelson, 2014; Fischer-Kern et al., 2015). Despite the theoretical rationale linking pretreatment RF with the establishment and development of the alliance, findings from the limited empirical research on the predictive validity of RF for the alliance have been inconsistent. In a study of a brief psychological treatment for patients with BPD, better metacognitive capacity—a construct overlapping with RF—had a positive predictive effect on therapist-rated but not on patient-rated alliance (Dimaggio et al., 2019). A study of MBT for BPD administered MASC (Movie for the Assessment of Social Cognition) pretreatment to assess social cognition (Kvarstein et al., 2020). Unexpectedly, hypermentalizing (excessive and flawed mentalizing) was associated with greater improvement of alliance over time, while hypomentalizing (reduced capacity to understand and reflect on one's own and others‘ mental states) did not impact alliance development. Regarding other conditions than BPD, results are also inconsistent. In a depression study (Ekeblad et al., 2016), two types of RF measures were applied, i.e., depression-specific RF (DSRF) and global RF (RF). Both lower pretreatment DSRF and RF predicted lower therapist-rated working alliance during treatment. However, for patient-rated alliance, RF had no predictive effect, whereas lower DSRF predicted lower patient-rated alliance. In addition, a study of bulimia nervosa found that higher pretreatment RF significantly predicted better observer-rated alliance (Katznelson et al., 2020).

In sum, there is insufficient knowledge regarding how the therapeutic alliance develops in MBT programs for BPD with conjoint individual and group psychotherapy, and whether RF predicts this development. The primary aim of this study was to investigate the development of the therapeutic alliance in an outpatient MBT program for BPD addressing both the individual and group therapy. Our hypotheses were that the alliance in individual therapy would be in the medium to high range in the first part of treatment, whereas for group therapy the therapeutic alliance would be in the medium range, and for both modalities the alliance would gradually improve through the course of treatment. The secondary aim was to investigate the predictive value of RF for the therapeutic alliance. Our hypothesis was that a higher pretreatment level of RF would be associated with a more positive development of the therapeutic alliance in individual and group therapy.

## Materials and methods

### Design, sample, and treatment setting

The study has a longitudinal, observational design and was conducted from October 2019 to March 2025 as part of the project “*Reflective functioning and psychotherapy processes in MBT”* at Oslo University Hospital, in an outpatient clinic for patients with personality disorder (PD). It comprises baseline data from 47 patients, and longitudinal data from 42. Patients were referred to the clinic internally within the specialist mental health service. The outpatient clinic is part of the Norwegian Network for Personality Disorders (The Network), a cross-regional collaboration of PD treatment units (Pedersen et al., 2022).

### Treatment

After an initial diagnostic assessment, patients with BPD or significant BPD traits were offered treatment in the MBT program (Karterud and Bateman, 2010; Karterud, 2019, 2012). Patients with alcohol or substance dependency, autism spectrum disorder, cognitive impairment, bipolar disorder type I, schizoaffective disorder, and schizophrenia were not included in the MBT program. As a general rule, if any of these conditions developed or were uncovered during treatment, participants were withdrawn from the program and transferred to other, more appropriate services. The MBT format comprised conjoint individual and group therapies, i.e., the patients' individual therapist was different from their group therapists. The treatment program had a maximum duration of 3 years. MBT individual therapy was delivered as weekly sessions the first year, every other week the second year, and every 2–4 weeks the third year. MBT groups began with initial psychoeducation (MBT-introductory group, 11 sessions, Karterud, 2019), followed by allocation to weekly group therapy (MBT-G). The groups were led by two therapists and consisted of up to eight patients in a slow-open format, with new patients joining as others left the group. The group sessions were structured according to the MBT manual (Karterud, 2012), with a main focus on exploration of interpersonal incidents aiming at improving the mentalizing capacity of the group members. These incidents should preferably be recent and in close relationships, and the group members brought them into the group in a turn-taking fashion.

At the start of treatment, an MBT case formulation was collaboratively developed by the patient and individual therapist, focusing on the patient's tasks and goals of therapy and potential challenges in the therapy process. The case formulation was revised in the individual therapy sessions as needed during the treatment (Karterud and Bateman, 2010; Bateman and Fonagy, 2016; Karterud and Kongerslev, 2019). MBT therapists collaborated closely in a treatment team with regular case discussions and weekly group-based supervision of video-taped individual and group therapy sessions, in accordance with MBT manuals (Karterud and Bateman, 2010; Karterud, 2019, 2012; Kvarstein et al., 2025). To facilitate therapist-patient collaboration, “mini-team” meetings between patient, individual and group therapists were held regularly to evaluate process and progress, with additional meetings convened when therapy was threatened by interpersonal challenges.

### Therapist qualifications and MBT fidelity

The majority of therapists were clinical psychologists, psychologists in postgraduate training, and psychiatrists. Other professions included one resident doctor, two social workers, one psychiatric nurse, and one occupational therapist. Basic knowledge of and training in MBT was emphasized. Eight were qualified MBT therapists, five had advanced training in psychodynamic psychotherapy, and eight had advanced training in group psychotherapy. Several therapists held more than one of these qualifications.

MBT fidelity of the individual therapists was assessed 6 months into treatment by an expert-rater affiliated with the Quality Laboratory of Psychotherapy. A total of 11 video-recorded individual therapy sessions conducted by 10 therapists were assessed based on the MBT Adherence and Competence Scale (Karterud et al., 2013). The scale ranges from 1 to 7, adherence and competence are scored separately, and a score of 4 or higher indicates adequate levels. Mean MBT adherence was 4.3 (SD = 0.8) and mean MBT competence was 4.5 (SD = 0.9). In this study, MBT fidelity for group therapists was not assessed. However, in a period prior to the study period, MBT fidelity was assessed and found satisfactory both for individual and group therapists (Kvarstein et al., 2020; Folmo et al., 2017).

### Pretreatment assessment of diagnoses

Diagnostic evaluation was performed according to DSM-5 (APA, 2013) by the clinicians at the outpatient clinic. All diagnoses were verified by a specialist in psychiatry or clinical psychology. Symptom disorders were assessed with the Mini-International Neuropsychiatric Interview (M.I.N.I.; Sheehan et al., 1994), and PDs with the Structured Clinical Interview for Section II DSM-5 Personality Disorders (SCID-5-PD; First et al., 2016).

### Repeated assessments of therapeutic alliance

As part of common routines in the Network, patients filled in a battery of questionnaires in the assessment phase and every 6 months during treatment. Measures of alliance were applied from assessment point 6 months and onwards until treatment termination.

For individual therapy, the short and revised version of the Working Alliance Inventory was applied (WAI-SR; Bordin, 1979; Hatcher and Gillaspy, 2006). This measure has twelve items rated on a 7-point Likert scale (score 1: “Never,” score 4: “Several times,” score 7: “Always”) and comprises the three subscales Goal, Task, and Bond. They address, respectively, agreement on treatment goals, collaboration on tasks in therapy, and the patient's emotional bond with the therapist. Each subscale has four items. This study used a mean score for each subscale, and by convention a score of 4 or higher indicated satisfactory alliance. WAI-SR has demonstrated good psychometric properties and is widely used in research and clinical settings (Munder et al., 2010).

For the assessment of therapeutic alliance in group therapy, the Group Questionnaire (GQ) was used (Krogel et al., 2013; Krogel, 2008; Pedersen et al., 2023). GQ has 30 items rated on a 7-point Likert scale (in the Norwegian version score 1: “Strongly disagree,” score 4: “Neither agree nor disagree,” score 7: “Strongly agree”). GQ has three domains: Positive Bond (PB), Positive Work (PW), and Negative Relationship (NR). PB measures group climate, alliance, and group cohesion, PW the agreement on therapeutic tasks and goals, whereas NR measures experience of conflict, alliance rupture, and empathic failure. Moreover, the domains have subscales, eight altogether, reflecting the tripartite relationship structure in group therapy, i.e., member-group, member-therapist(s), and member-member. See Figure 1 for description of GQ subscales. This study used a mean score for each domain and subscale, and according to the scale a score of 4 represents a neutral response. For PB and PW and their subscales, higher scores indicate better group relationships, whereas the scale is reversed for NR and its subscales. GQ norms for different clinical populations are not established (Janis et al., 2018). In the present study, scores 5 or higher for PB and PW and their subscales, and 3 or lower for NR and its subscales were interpreted as satisfactory therapeutic alliance. GQ has demonstrated satisfactory psychometric properties, also in PD samples (Pedersen et al., 2023; Janis et al., 2018; Giannone et al., 2020; van den Heuvel et al., 2025).

**Figure 1:**
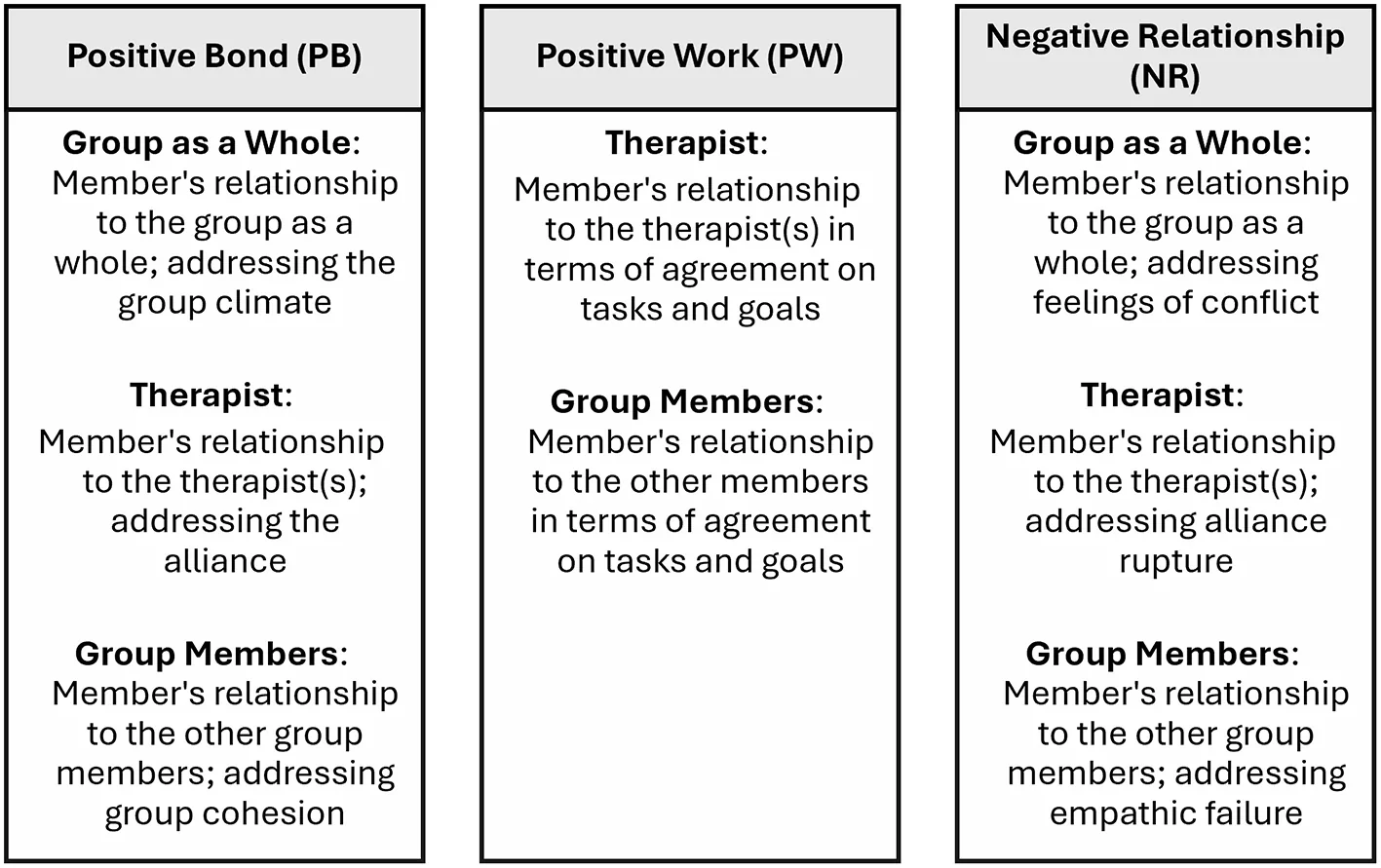
Group questionnaire (GQ) domains and subscales.

### Assessment of reflective functioning

Two interviews were applied to assess RF in the initial evaluation. The Adult Attachment Interview (AAI; George et al., 1985) was conducted by two specially trained researchers (TW & external researcher). This is a semi-structured interview lasting approximately one hour, addressing the individual's experiences of childhood relationships with the primary caregivers and their influences on the individual as an adult. Verbatim transcripts of the interviews were scored using the Reflective Functioning Scale (RFS) resulting in a global RF score (Fonagy et al., 1998). RFS is an eleven-point scale ranging from −1 (anti-reflective) to + 9 (exceptional reflective). Further details on scoring procedure are described in a previous publication (Ulvestad et al., 2024). RF based on the AAI (AAI-RF) is considered the gold standard for RF assessment and has shown to be a valid and reliable measure of RF also in BPD-samples (Bouchard et al., 2008; Katznelson, 2014; Taubner et al., 2013). In the present project, interrater reliability for the two AAI-RF raters was estimated using the Intra-class Correlation Coefficient (ICC). The ICC was 0.85 (95% CI: 0.27–0.97), indicating good reliability (Ulvestad et al., 2024; Koo and Li, 2016).

The Mentalization Breakdown Interview (MBI; Ulvestad et al., 2023) is a recently developed RF assessment method that the clinicians conducted as part of the initial assessment. This semi-structured 30-min interview explores a recent and preferably severe episode of mentalizing breakdown in a close relationship. Based on video recording of this exploration, independent certified RF-raters assigned an RF score (MBI-RF) using the RFS (Fonagy et al., 1998). Further details on the MBI and scoring procedure are described in previous publications (Ulvestad et al., 2024, 2023), which also provide preliminary support for the MBI as a reliable and valid BPD-specific RF assessment method. The ICC for the three MBI-RF raters was 0.81 (95% CI: 0.61–0.92), indicating good reliability.

### Other descriptive data

Patient self-report on demographics, former treatment experience, and former (lifetime) experiences of self-harming and suicidal acts was obtained during the initial assessment.

Personality functioning was assessed with the Level of Personality Functioning Scale-Brief Form, second version (LPFS-BF 2.0; Weekers et al., 2019), a patient self-report based on the Level of Personality Functioning Scale in DSM-5 AMPD (Alternative Model of Personality Disorders, Section III; APA, 2013). LPFS-BF has 12 items clustered in the two domains self-functioning (Self) and interpersonal functioning (Other). Two different response formats exist for LPFS-BF 2.0, and in this study the 0–3 format was applied. The sum score of Self and Other is reported (LPFS-BF Total), where the thresholds indicating, respectively, mild, moderate, severe, and extreme personality dysfunction are 14, 19, 24, and 29 (Weekers et al., 2023; Hualparuca-Olivera et al., 2025).

Depressive symptoms were measured with the Patient Health Questionnaire, Depression (PHQ-9; Kroenke et al., 2001), and generalized anxiety with the Generalized Anxiety Disorder-7 (GAD-7; Spitzer et al., 2006). These self-report measures apply a four-point (0–3) Likert scale and sum scores ≥ 10 indicate clinically relevant symptoms (Ettman et al., 2020; Kroenke et al., 2007).

Social and occupational functioning was assessed with the Work and Social Adjustment Scale (WSAS; Mundt et al., 2002), a self-report questionnaire with five items rated on a nine-point Likert scale (0–8). Sum scores define impairment levels as follows: mild-to-no (< 15), moderate-to-severe (15–30), and extreme (>30; Mataix-Cols et al., 2005). The clinicians assessed psychosocial functioning using the Global Functioning Scale (GFS; Pedersen et al., 2018). The GFS gives scores (range 0–100) of both symptom severity and social impairment, where the lower of the two scores is reported here. Clinical levels are by convention indicated by GFS < 60.

## Participants

Sociodemographic and clinical status at pretreatment assessment is presented in Table 1. The vast majority of the patients were females in a young adult group (M = 23.2, SD = 3.3; range 19–30 years). Scores on GFS and WSAS indicated moderate to severe impairment of social functioning, as did scores reflecting symptom distress (PHQ-9 and GAD 7). The LPFS-BF sum score confirmed impaired personality functioning at a level indicating moderate personality dysfunction. The mean level of RF was 2.3 (SD = 1.0) for AAI and 2.0 (SD = 1.0) for MBI. According to the RFS, these levels fall between absent RF (RF = 1) and questionable RF (RF = 3), indicating significant problems with mentalizing at a level typically seen in patients with severe personality pathology. RF at this level indicates that the patient has marked difficulty understanding their own and others' behavior in terms of inner thoughts and feelings, instead relying on concrete explanations, clichés, and rigid assumptions (Fonagy et al., 1998). Clinically, this makes the patient highly vulnerable to misinterpreting others' intentions and feeling easily rejected or mistreated, which, under emotional or relational stress, often leads to rapid escalation of distress, conflicts, and impulsive or dysregulated reactions (Luyten et al., 2020). The RF scores were in the range 0–5 for both measures, with the majority of scores in the range 1–3. The vast majority had former experience of self-harm, 36% started before age 13 years, and 68% reported self-harm last 6 months, 26% daily or weekly last 6 months. Most patients reported considerable former experience of treatment within mental health services, and 45% had previous hospital admissions.

**Table 1:** Demographic and clinical status at initial assessment.

	Percent	Mean (SD)
Demographics		
Age		23.2 (3.3)
Female	87	
Cohabiting or married	19	
Years education after mandatory school (age 6–16)		3.8 (2.4)
Months > 50% work/study last 6 months		3.0 (2.8)
Former treatment experience		
Previous treatment in mental health services	87	
More than two treatment series	51	
First treatment < 18 years of age	75	
Previous hospital admissions	45	
Functioning		
Global functioning (GFS)		51.7 (4.6)
Work and social impairment (WSAS)		24.4 (6.7)
Level of personality functioning (LPFS-BF total)		21.9 (5.2)
Reflective functioning		
AAI-RF		2.3 (1.0)
MBI-RF		2.0 (1.0)
Symptom distress		
Depression (PHQ-9)		20.7 (3.6)
Anxiety (GAD-7)		13.7 (5.0)
Self-harming and suicide attempts		
Self-harm, lifetime	89	
Self-harm < 13 years of age	36	
Self-harm, last 6 months	68	
Self-harm, daily or weekly last 6 months	26	
Suicide attempt, lifetime	62	
Suicide attempt < 13 years of age	4	
Suicide attempt, last 6 months	13	

This table presents demographic and clinical status at initial assessment (*n* = 47).

GFS, Global Functioning Scale; WSAS, Work and Social Adjustment Scale; LPFS-BF, the Level of Personality Functioning Scale-Brief Form; AAI-RF, Reflective Functioning scored from the Adult Attachment Interview; MBI-RF, Reflective Functioning scored from the Mentalization Breakdown Interview; PHQ-9, Patient Health Questionnaire, Depression; GAD-7, Generalized Anxiety Disorder-7.

Diagnostic status is presented in Table 2. In line with the target group for the MBT program, 83% had a BPD diagnosis, and 91% four or more fulfilled BPD criteria. Mean number of BPD criteria was 6.0 (SD = 1.8). Avoidant PD (AvPD) was the most common co-occurring PD. All patients except one had one or more PD diagnoses (81% had one PD, 11% two, and 6% three). Mean number of SCID-5-PD criteria was 13.5 (SD = 5.2). The mean number of other mental disorders was 1.2 (SD = 1.4). Almost one third of the patients qualified for a current diagnosis of major depression, with PTSD (19%) being the next most prevalent comorbid mental disorder.

**Table 2:** Diagnostic status at initial assessment.

	Percent	Mean (SD)
Personality disorders		
Schizoid & schizotypal	0	
Paranoid	2	
Antisocial	0	
Narcissistic & histrionic	0	
Borderline	85	
Avoidant	17	
Dependent	4	
Obsessive compulsive	4	
PD NOS	9	
Severity of personality difficulties		
Total number of SCID-5-PD criteria		13.5 (5.2)
Number of BPD criteria		6.0 (1.8)
Number of PD diagnoses		1.2 (0.6)
Mental disorders		
Number of diagnoses		1.2 (1.4)
Major depression	32	
PTSD	19	
Agoraphobia with panic disorder	13	
Eating disorder NOS	11	
GAD	6	

This table presents diagnostic status for patients at initial assessment (n = 47).

PD NOS, personality disorder not otherwise specified; PTSD, post-traumatic stress disorder; GAD, generalized anxiety disorder.

## Statistics

All analyses used SPSS Statistics for Windows, Version 30 (IBMCorp, 2024). Linear mixed models (LMM) based on maximum likelihood statistics were the main statistical method (Heck et al., 2010; Singer and Willett, 2003). All models applied the continuous time-variable “months from first assessment.” Time was coded such that 0 corresponded to the first assessment of alliance, i.e., at 6 months. The estimated intercepts thus reflect levels at that time. To assess the model fit, log-likelihood ratio test (-2LL) and Akaike Information Criterion (AIC) were used, where smaller is better.

## Longitudinal analyses

The primary aim of the study was addressed using LMM, with patient self-reports of the therapeutic alliance as dependent variables, i.e., the three subscales of WAI-SR (Goal, Task, and Bond), and for GQ the three domains (PB, PW, and NR) and the eight subscales described above. Thus, altogether fourteen linear change models for the dependent variables. For all except one, the best-fitted models included linear time, random intercept and slope, and unstructured covariance. Due to lack of model convergence, the best-fitted model for the dependent variable Group as a whole-subscale of GQ NR included linear time, random intercept, fixed slope, and covariance structure Identity. For this model, variation in slopes was therefore not estimated.

Longitudinal, 6–30 month effect sizes (ES) were based on LMM predicted values. They were calculated according to Cohen's *d*; small ES: *d* = 0.2, medium *d* = 0.5, large *d* = 0.8 (Cohen, 1988). Six months was the first assessment point for the therapeutic alliance, and 30 months was the assessment point closest to mean treatment duration (27 months).

The secondary aim of the study was addressed by adding AAI-RF and MBI-RF as baseline predictors in the WAI-SR and GQ models given above.

## Sample size and number of repeated measurements

For the longitudinal analyses a subsample of 42 was applied, in which all participants had repeated assessments of WAI (M = 3.6, SD = 1.8) and GQ (M = 3.2, SD = 1.8). 75% had three or more WAI assessments, and 66% had three or more GQ assessments.

## Missing and unbalanced data

In this naturalistic design, missing assessments may be due to multiple factors. Generally, an important cause of missing data are errors in the administration of paper-based questionnaires. To investigate potential bias from missing assessments on the development of the therapeutic alliance, variables counting the number of WAI and GQ assessments were included as predictors in separate models for the dependent variables (Hedeker and Gibbons, 1997; Kvarstein et al., 2023). In these models, the number of assessments did not predict deviating change patterns in the dependent variables (*p* > 0.05).

Although the MBT program had a recommended long-term duration, actual treatment duration varied within the sample, and shorter treatment necessarily resulted in fewer assessments. Treatment duration was therefore also investigated as a longitudinal predictor in separate models for all dependent variables. In these models, treatment duration was not associated with deviating longitudinal change in any of the WAI or GQ variables (*p* > 0.05).

## Results

### Treatment duration and dropout

Mean treatment duration was 27 months (SD = 13), with a median of 31 (range 0–45). Early dropout, defined as treatment lasting less than 6 months irrespective of reason, was 6%. Overall, 87% remained in treatment for at least 12 months, whereas 65% remained for 18 months or more.

## Therapeutic alliance in individual therapy

As presented in Table 3 and Figure 2, LMM analyses demonstrated satisfactory levels of alliance (≥ 4) in individual therapy at the first assessment. For all three subscales of WAI the levels were above five (5.1–5.4) in this early phase. Regarding the monthly rate of change, the Bond level was generally stable, though trending toward minor improvement over time (*p* = 0.086), with a 6–30-month ES of *d* = 0.19. For Task and Goal, slope increase was small but statistically significant (*p* = 0.011 and *p* = 0.028, respectively), with 6–30-month LMM-based ES of *d* = 0.20 and *d* = 0.26. According to variance components in LMM, there was no significant longitudinal between-patient variation for Bond and Task, whereas for Goal there was a significant but minimal longitudinal between-patient variation.

**Figure 2:**
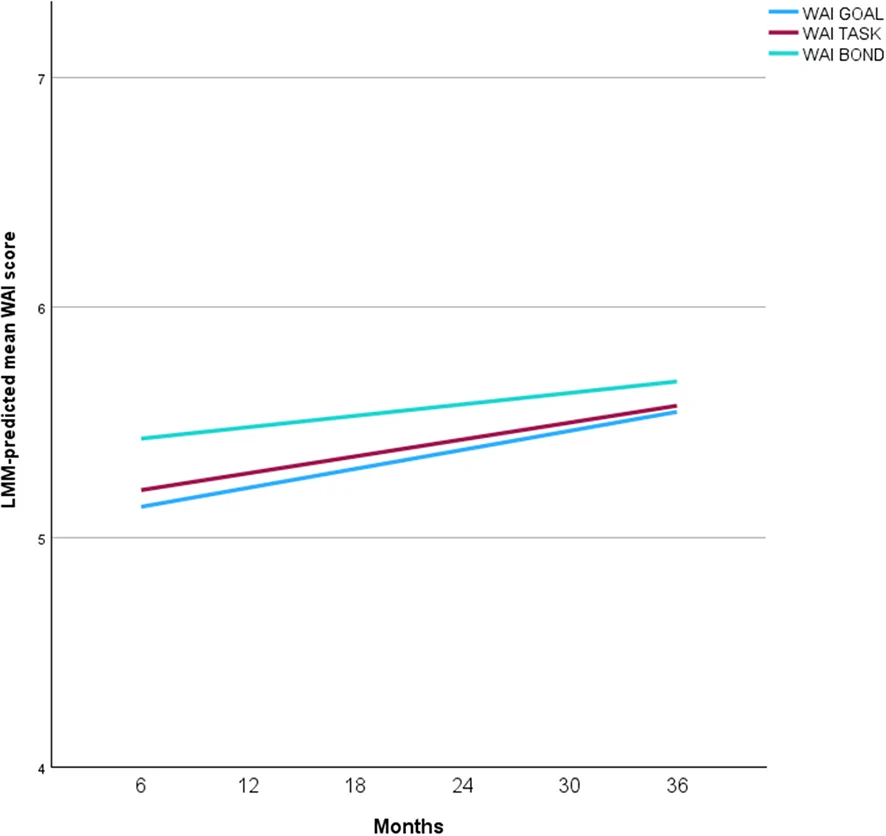
Therapeutic alliance development: WAI scores. Figure based on LMM-predicted mean WAI scores at each assessment point.

**Table 3:** WAI—longitudinal trajectories and relation to reflective functioning.

Model specification	Predictors	Fixed effects: estimates for linear trajectories				Variance components		Model Fit	
		Intercept (SE)	*p*	Slope (SE)	*p*	Intercept (SE)	Slope (SE)	−2LL	AIC
**WAI Bond**		5.38 (0.21)	< 0.001	0.008 (0.005)	0.086	1.34 (0.40)^**^	0.00 (0.0)	490	502
Separate predictor models	AAI-RF	−0.019 (0.21)	0.930	0.007 (0.005)	0.125	0^▴^	0^▴^		503
	MBI-RF	−0.22 (0.20)	0.284	0.007 (0.005)	0.161	3^▴^	0^▴^		503
**WAI Task**		5.13 (0.25)	< 0.001	0.012 (0.005)	0.011	2.26 (0.57)^**^	0.00 (0.0)	469	481
Separate predictor models	AAI-RF	0.24 (0.25)	0.346	−0.001 (0.005)	0.795	3^▴^	0^▴^		484
	MBI-RF	0.04 (0.24)	0.875	−0.003 (0.005)	0.578	0^▴^	0^▴^		485
**WAI Goal**		5.05 (0.23)	< 0.001	0.014 (0.006)	0.028	1.88 (0.49)^**^	0.001 (0.0)^**^	498	510
Separate predictor models	AAI-RF	0.27 (0.24)	0.272	−0.005 (0.006)	0.446	3^▴^	0^▴^		513
	MBI-RF	0.15 (0.23)	0.519	−0.011 (0.006)	0.073	5^▴^	0^▴^		511

This table presents LMM estimates (*n* = 42) of baseline levels (intercept) and monthly rates of change (slope) for three dependent variables, separate predictor analyses, and variance estimates. Significant variance estimates are indicated by ^*^p < 0.05 and ^**^p < 0.01. ^▴^ indicates percent explained variance; the open model (without predictors) is used as the reference model. Model fit is indicated by the log-likelihood ratio statistic (−2LL) and Akaike Information Criterion (AIC), where smaller is better.

## Therapeutic alliance in group therapy

LMM analyses of the three GQ domains and its eight subscales are presented in Table 4, whereas the longitudinal trajectories of the eight subscales are illustrated in Figure 3.

**Figure 3:**
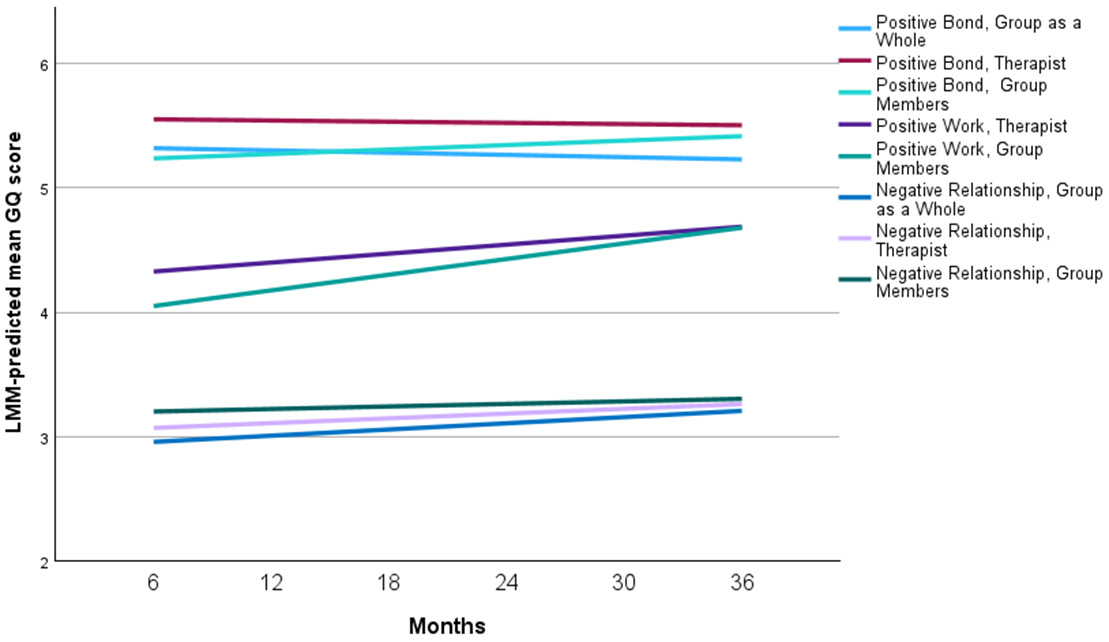
Therapeutic alliance development: GQ scores. Figure based on LMM-predicted mean GQ scores at each assessment point. For the Negative Relationship subscales, the response scale is reversed.

**Table 4:** GQ—longitudinal trajectories and relation to reflective functioning.

Model specification	Predictors	Fixed effects: estimates for linear trajectories				Variance components		Model fit	
		Intercept (SE)	*p*	Slope (SE)	*p*	Intercept (SE)	Slope (SE)	−2LL	AIC
**Positive Bond (PB)**		5.36 (0.19)	< 0.001	0.001 (0.005)	0.908	1.07 (0.32)^**^	0.00 (0.0)	379	391
Separate predictor models	AAI-RF	0.07 (0.19)	0.713	0.004 (0.005)	0.432	1^▴^	0^▴^		394
	MBI-RF	−0.07 (0.18)	0.690	−0.003 (0.004)	0.530	0^▴^	0^▴^		394
PB—group as a whole		5.34 (0.18)	< 0.001	−0.003 (0.005)	0.553	0.86 (0.30)^**^	0.00 (0.0)	399	411
Separate predictor models	AAI-RF	0.03 (0.19)	0.866	0.003 (0.005)	0.523	1^▴^	0^▴^		414
	MBI-RF	−0.08 (0.18)	0.642	−0.003 (0.005)	0.569	1^▴^	0^▴^		414
PB—therapist		5.56 (0.23)	< 0.001	−0.002 (0.005)	0.755	1.64 (0.48)^**^	0.00 (0.0)	429	441
Separate predictor models	AAI-RF	0.09 (0.24)	0.713	0.003 (0.005)	0.518	1^▴^	0^▴^		444
	MBI-RF	−0.06	0.774	−0.006 (0.005)	0.213	0^▴^	0^▴^		443
PB—group members		5.20 (0.21)	< 0.001	0.006 (0.006)	0.324	1.25 (0.40)^**^	0.001 (0.0)^*^	431	443
Separate predictor models	AAI-RF	0.11 (0.22)	0.618	0.004 (0.006)	0.553	2^▴^	0^▴^		446
	MBI-RF	−0.07 (0.21)	0.727	0.000 (0.006)	0.962	1^▴^	0^▴^		447
**Positive Work (PW)**		4.09 (0.25)	< 0.001	0.016 (0.007)	0.019	2.00 (0.57)^**^	0.001 (0.0)^*^	461	473
Separate predictor models	AAI-RF	−0.04 (0.26)	0.869	0.005 (0.007)	0.497	0^▴^	0^▴^		477
	MBI-RF	−0.07 (0.25)	0.788	−0.002 (0.007)	0.711	0^▴^	0^▴^		477
PW—therapist		4.25 (0.26)	< 0.001	0.012 (0.007)	0.089	2.12 (0.62)^**^	0.001 (0.0)^*^	479	491
Separate predictor models	AAI-RF	−0.11 (0.27)	0.702	0.007 (0.007)	0.319	0.5^▴^	0^▴^		494
	MBI-RF	−0.10 (0.26)	0.707	−0.003 (0.007)	0.706	0^▴^	0^▴^		494
PW—group members		3.92 (0.26)	< 0.001	0.021 (0.007)	0.007	1.81 (0.58)^**^	0.001 (0.0)^*^	500	512
Separate predictor models	AAI-RF	0.02 (0.27)	0.942	0.002 (0.008)	0.779	0^▴^	0^▴^		516
	MBI-RF	−0.05 (0.25)	0.860	−0.002 (0.007)	0.763	0^▴^	0^▴^		516
**Negative Relationship (NR)**		3.05 (0.18)	< 0.001	0.006 (0.005)	0.217	0.92 (0.29)^**^	0.00 (0.0)	382	394
Separate predictor models	AAI-RF	−0.12 (0.18)	0.504	−0.004 (0.005)	0.444	3^▴^	0^▴^		396
	MBI-RF	−0.03 (0.18)	0.856	0.003 (0.004)	0.550	0^▴^	0^▴^		397
NR—group as a whole		2.91 (0.19)	< 0.001	0.008 (0.006)	0.145	0.55 (0.20)^**^		463	471
Separate predictor models	AAI-RF	−0.14 (0.19)	0.485	−0.002 (0.006)	0.744	5^▴^			473
	MBI-RF	−0.06 (0.19)	0.765	0.002 (0.006)	0.698	0^▴^			475
NR—therapist		3.03 (0.23)	< 0.001	0.006 (0.005)	0.210	1.62 (0.49)^**^	0.00 (0.0)	451	463
Separate predictor models	AAI-RF	−0.14 (0.24)	0.558	−0.005 (0.005)	0.372	2^▴^	0^▴^		466
	MBI-RF	0.03 (0.23)	0.894	0.003 (0.005)	0.510	0^▴^	0^▴^		466
NR—group members		3.18 (0.23)	< 0.001	0.003 (0.007)	0.610	1.39 (0.49)^**^	0.001 (0.0)	471	483
Separate predictor models	AAI-RF	−0.07 (0.24)	0.772	−0.006 (0.007)	0.400	2^▴^	0^▴^		486
	MBI-RF	−0.03 (0.23)	0.881	0.001 (0.006)	0.886	0^▴^	0^▴^		487

This table presents LMM estimates (*n* = 42) of baseline levels (intercept) and monthly rates of change (slope) for all dependent GQ variables, separate predictor analyses, and variance estimates. Significant variance estimates are indicated by ^*^p < 0.05 and ^**^p < 0.01. ^▴^ indicates percent explained variance; the open model (without predictors) is used as the reference model. Model fit is indicated by the log-likelihood ratio statistic (−2LL) and Akaike Information Criterion (AIC), where smaller is better.

For PB and the three subscales, the estimates for the first assessment were high (5.2–5.6), indicating satisfactory levels in the early phase of group therapy. The estimated slopes for PB and the subscales were not significant, indicating stable levels of group climate, alliance and group cohesion as the treatment progressed. The 6–30-month ES for the domain was *d* = 0.01, and for the subscales *d* = −0.10 (Group as a Whole), *d* = −0.03 (Therapist), and *d* = 0.14 (Group Members).

For PW and the two subscales, the estimates for the first assessment (3.9–4.3) were lower than PB and below the satisfactory range. The longitudinal improvement was however significant for the PW domain (*p* = 0.019), with a small-to-medium ES (*d* = 0.30). The estimate for the first assessments was 4.1 for the domain, and for the subscales, respectively, 4.3 (Therapist) and 3.9 (Group Members). The slope for PW Therapist did not reach statistical significance (*p* = 0.089), and the ES was small (*d* = 0.22). For PW Group Members, the longitudinal change was significant (*p* = 0.007) approaching a medium ES (*d* = 0.43), indicating an increasing agreement with the other group members on tasks and goals in therapy.

For NR, the scale is reversed, and levels above 3 can indicate unsatisfactory group relationships. The estimate for the first assessment was 3.0 for the domain, and for the subscales, respectively, 2.9 (Group as a Whole), 3.0 (Therapist), and 3.2 (Group Members). Thus, all estimates except one were within the satisfactory range. Despite small differences between the subscales, the estimate for Group Members indicated some challenges in the relationship with the other group members in the early phases of the treatment. Moreover, there was no significant longitudinal reduction for neither the NR domain, nor its subscales. The 6–30-month ES were, respectively, *d* = 0.15 (domain), *d* = 0.33 (Group as a Whole), *d* = 0.12 (Therapist), and *d* = 0.08 (Group Members). To summarize, there was no statistically significant improvement in terms of experience of conflict, alliance ruptures, or emphatic failures in the group relationships during treatment.

According to variance components in LMM, there were no significant longitudinal between-patient variation for the domains PB and NR, whereas for PW there was a significant but minimal longitudinal between-patient variation.

## Predictive validity of reflective functioning

The separate models with AAI-RF and MBI-RF as predictors are presented in Tables 3, 4. None of the predictor models improved model fit. Neither AAI-RF nor MBI-RF at baseline had significant effects on intercept or slope for WAI or GQ with its subscales.

## Discussion

This study examines the development of the therapeutic alliance in an outpatient MBT program for BPD, focusing on both the individual and group psychotherapy, and whether RF predicts the therapeutic alliance in this context. The patients in this study presented with substantial symptom severity, low levels of RF, and marked functional impairment, typical of MBT target populations. Overall, the findings indicate satisfactory levels of therapeutic alliance in the MBT program. The main findings will be discussed with an emphasis on clinical implications.

## Sustained satisfactory therapeutic alliance in individual therapy

Consistent with our hypothesis, initial levels of alliance in the individual therapy were in the medium to high range, with Bond, Task, and Goal subscale levels comparable to those reported by Folmo et al. (2021) in mentalization-based individual therapy and to the satisfactory initial alliance levels found in DBT for BPD (Barnicot et al., 2016; Bedics et al., 2015). In our study, agreement on tasks and goals improved slightly over the course of treatment, whereas, contrary to our expectation and the study of Folmo et al. (2021), the affective bond remained stable, with only a trend toward minor improvement over time. Yet, in Barnicot et al. (2016), alliance levels were stable over the course of treatment, whereas in Bedics et al. (2015) they gradually increased toward the end of treatment, also for the subscale assessing the affective bond. The repeated scores on Bond in our sample tended to cluster near the top of the scale, which raises the possibility of a ceiling effect (Meier, 2022; Paap et al., 2020). Nevertheless, given the target population, alliance levels in individual therapy were notably high, indicating that the MBT program effectively established and maintained a satisfactory alliance in this modality.

## Some challenges to the therapeutic alliance in group therapy

Regarding the therapeutic alliance in the group therapy, the findings deviated from the hypothesis in several respects. Initial levels of PB were higher than expected, indicating that the MBT program facilitated early bonding in the group, and positive bonding remained stable throughout treatment. Hence, even if the expected improvement did not occur, the high initial levels indicate sustained satisfactory bonding. In contrast, initial levels of PW and its two subscales (agreement on tasks and goals) were below the satisfactory range. Interestingly, over the course of treatment this improved at the domain level and in relation to the other group members, with small to medium ES, but not in relation to the therapists. Thus, even though MBT has a framework intended to facilitate agreement on tasks and goals, such as the MBT case formulation, the findings suggest that the group therapists are not sufficiently involved in this process.

Regarding the NR domain (i.e., experience of conflict, alliance rupture, empathic failure), initial estimates were clustered near the threshold yet remained within the satisfactory range, except for the relationship with the other group members, where there were indications of some challenges in the early phase of treatment. Given the lack of longitudinal improvement in this domain and its subscales, these somewhat strained relationships with other group members appear to have persisted throughout treatment. This could, however, be viewed as acceptable in a treatment program intended for a patient group typically characterized by significant interpersonal difficulties. The finding is particularly noteworthy given the slow-open group format, which involves substantial turnover of group members over the course of treatment. Despite its distressing potential, this turnover may serve a useful function by providing continuous exposure to new interpersonal situations and dynamics (Barrett et al., 2025). Thus, a certain level of interpersonal strain is consistent with the MBT framework, which emphasizes the value of rupture-repair processes in group therapy as a “training ground” for mentalizing and healthier interpersonal functioning, provided that the therapists carefully facilitate the process to help patients tolerate and work through difficult experiences (Barrett et al., 2025; Bateman et al., 2021; Barnicot et al., 2022). Notably, despite relational distress, positive bonding in the group was maintained. Future research should investigate how such experiences impact treatment outcome.

## Building the therapeutic alliance in MBT

It was beyond the scope of this study to disentangle the relative effectiveness of the various MBT components to the therapeutic alliance, and future studies in this field are warranted. Yet, certain components in the MBT program stand out as potentially important for the alliance, several of these in accordance with recommendations from a large meta-analytic synthesis on alliance in psychotherapy (Fluckiger et al., 2018). For instance, the MBT case formulation may be essential for establishing an early and sustained positive alliance (Fluckiger et al., 2018). In a study by Kramer et al. (2023), treatment based on case formulation predicted session-by-session increases in therapeutic alliance in the treatment for BPD, though only for therapist-rated alliance. The case formulation in the MBT program specifies the patient's tasks and goals in therapy, attachment strategies under stress, emotion regulation difficulties, mentalizing impairments, and other potential obstacles in the treatment process, thereby addressing core aspects of the therapeutic alliance and potential challenges (Karterud and Kongerslev, 2019). However, the suboptimal levels of agreement on tasks and goals in the groups, particularly between group members and group therapists, suggest that the full potential of the case formulation has not been realized in the program. This is in line with previous calls to strengthen the MBT case formulation's role in MBT-G, for example by reading it aloud and discussing it regularly in the groups (Karterud, 2018). Another proposal is the preparation and use of an MBT Passport, i.e., a short version of the case formulation that each patient presents in the group on a regular basis (Grove and Smith, 2022).

The initial psychoeducative group (MBT-I) may also serve as an alliance-promoting component (Stoffers-Winterling et al., 2022; Zanarini et al., 2018). This aligns with Wampold and Imel's summary of psychotherapy research, which concludes that psychoeducation—including the explanation of the treatment rationale—can be considered one of the common factors in psychotherapy (Wampold and Imel, 2015). MBT-I provides psychoeducation on the concepts of mentalizing, attachment, emotions and feelings, and the core features of PD and BPD, as well as a detailed presentation of the MBT program. This may help socialize patients to the treatment model, clarify treatment expectations, and foster more realistic views of the course and outcome of therapy (Barrett et al., 2025; Ditlefsen et al., 2021).

Regular supervision is a key element in MBT. The video-based supervision of both treatment modalities in the present study emphasized explicit work on the relationships between patients and therapists, including transference and countertransference (Karterud and Bateman, 2010; Karterud et al., 2020). In the treatment of patients with BPD, therapists frequently report feeling overwhelmed, incompetent or inadequate, and confused and frustrated in sessions, potentially compromising their therapeutic performance (Colli et al., 2014). A recent study found significant associations between early negative countertransference and lower patient-rated alliance in a PD sample, which remained stable throughout therapy (Breivik Øvstebø et al., 2024). Supervision may increase therapists' empathy and understanding of patients' difficulties and facilitate the timely and explicit addressing of alliance ruptures, which may have contributed to the positive bond with therapists in both modalities (Fluckiger et al., 2018; Safran and Muran, 2000). Similarly, the MBT structure of regular and extraordinary “mini-team” meetings involving the patient and therapists may be an important means of addressing alliance ruptures in both treatment modalities. However, given the unsatisfactory levels of agreement on tasks and goals between patients and group therapists in the present study, such meetings may have an unrealized potential for fostering clearer and more mutual agreement in this respect.

Furthermore, several specific interventions in MBT are intended to promote patient engagement, active participation, and a sense of mastery in the therapeutic process. Such interventions may strengthen the alliance, as highlighted in quantitative and qualitative research on early alliance formation (Lavik et al., 2018; Sexton et al., 2005). For both treatment modalities, salient interventions include the emphasis in MBT on tailoring therapeutic interventions to the patient's current level of RF, modulating affective arousal to an optimal level (neither too high nor too low) during sessions, and therapists' “not-knowing” stance rather than an expert stance (Karterud and Bateman, 2010).

In MBT-G for BPD, the group-specific interventions addressing the structure are of particular importance, especially given the risk of conflicts and destructive acting out that may seriously affect group relationships (Bateman and Fonagy, 2016). In this context, the intervention “Managing authority” is vital, underscoring the importance of providing the group with structure and guidance and of therapists being more explicitly responsible for the therapeutic process compared with traditional group analysis (Karterud, 2015). In MBT-G, however, therapists aim to obtain an optimal balance between providing structure and allowing sufficient freedom for exploring and testing interpersonal dynamics that may catalyze therapeutic growth (Barrett et al., 2025). The findings in this study indicate subtle but persistent challenges in relationships with fellow group members, which do not appear to worsen over time, and this pattern could be consistent with such a balance. In this context, the explicit focus in individual therapy on exploring and mentalizing interpersonal challenges arising in the group is also likely an important factor supporting ongoing rupture-and-repair processes in the group (Karterud and Bateman, 2010; Bateman et al., 2021).

## Notably low early dropout and high patient retention in the MBT program

High rates of dropout have been widely reported in evidence-based psychotherapies for BPD, and systematic reviews indicate that the therapeutic alliance is a critical protective factor against dropout (De Salve et al., 2025; de Freixo Ferreira et al., 2023). The findings in our study are consistent with this: overall satisfactory alliance levels in the MBT program were accompanied by a low rate of early dropout (6 %), with 87% remaining in treatment for at least 12 months and 65% for 18 months or more. These treatment retention figures are consistent with a meta-analysis of treatment completion in psychotherapy for BPD, which reported an overall completion rate of 75 % for interventions lasting less than 12 months and 71 % for longer interventions (Barnicot et al., 2011). However, in our study, the reasons for treatment termination were not recorded. We were therefore unable to distinguish between “true” dropout and other reasons for discontinuation or planned termination, such as referral to other specialized treatments for comorbid conditions.

## No predictive validity of reflective functioning for therapeutic alliance

Initial levels of RF were low across both assessment methods (Fischer-Kern et al., 2015; Keefe et al., 2023). Our hypothesis that higher pretreatment RF would be positively associated with the development of therapeutic alliance in individual and group therapy was not supported for neither AAI-RF nor MBI-RF, the latter being a more BPD-specific RF measure (Ulvestad et al., 2023). Except for the study of Jørgensen et al. (2021) which found that lower RF predicted dropout in MBT-G among adolescents with BPD, no previous study has investigated the significance of pretreatment RF in MBT. A possible explanation for the present finding is the emphasis in MBT on therapists' continuously adjusting their overall approach and specific interventions to patients' current RF level, thereby potentially ensuring a satisfactory alliance across patients with different RF levels (Karterud and Bateman, 2010; Bateman and Fonagy, 2016). This aligns with a recent review by Luyten et al. (2024) on the role of RF in psychological interventions in adults, which suggests that pre-existing mentalizing impairments might be significant only if they are insufficiently addressed during therapy. Still, RF assessed with the RFS yields a global score, rather than a differentiated assessment of specific mentalizing capacities (Hüwe et al., 2023; Choi-Kain and Gunderson, 2008), and may therefore fail to capture specific facets of RF of relevance for the alliance. In addition, the relatively homogeneous development of the alliance in our sample, with no or only minimal longitudinal between-patient variation, together with the narrow range of RF scores, may also have contributed to the absence of significant predictive effects. Moreover, in the present study, the first assessment of therapeutic alliance was conducted at 6 months, which may have limited the detection of any early influence of pretreatment RF on alliance formation. We do not know whether RF changed during treatment and possible implications of such changes for the alliance. Future studies employing repeated assessments of RF are needed to clarify how such changes affect alliance development.

## Strengths and limitations

A major strength of the study is its high ecological validity due to its naturalistic design. The treatment was delivered under routine clinical conditions in a PD outpatient clinic, involving a clinically representative sample of patients with moderate to severe BPD.

Nevertheless, several limitations should be considered when interpreting the findings. First, owing to the observational design and lack of a control group, causal conclusions cannot be drawn. We are not able to determine whether the high levels of therapeutic alliance were caused by MBT *per se* rather than by common therapeutic factors. Second, the relatively small sample size (*n* = 42) introduces the risk of Type II errors. However, observer-based RF (such as AAI-RF and MBI-RF) is labor-intensive, and psychotherapy studies involving RF assessment are thus often small-sampled. In a review of observer-based and computerized measures of patients' mentalization in psychotherapy, more than 60% of the studies had a sample size of 30 or fewer (Hüwe et al., 2023). When performing LMM analyses, sample sizes of at least 100 are often preferred, but LMMs can be fitted with smaller samples, and the number of repeated measures per participant also plays an essential role in model estimation and statistical power (Curran et al., 2010). In the present study, the majority of participants had three or more assessments, and this longitudinal data quality increased statistical power despite the modest sample size. Yet, a large number of LMM analyses were conducted without any correction for multiple testing, thereby increasing the risk of Type I errors, while avoiding an undue increase in type II errors.

Third, due to standard routines at the outpatient clinic, the first assessment of therapeutic alliance was not conducted until 6 months into treatment. The possibility of significant changes in alliance prior to this time point cannot be ruled out. However, in the earlier study by Folmo et al. (2021) conducted in the same clinic, WAI levels at 3 months were comparable to the levels at 6 months in this study, which provides additional support for the establishment of a satisfactory alliance early in the MBT program. Yet, for some participants in our study, the first assessment of alliance coincided with participation in the initial psychoeducative group, complicating interpretation of the initial levels of therapeutic alliance in MBT-G. Moreover, patients' self-report forms (WAI and GQ) were distributed by the individual therapist, and the scores were reviewed in the individual therapy sessions. This may have contributed to inflated alliance scores, probably most pronounced for WAI, given that patients may wish to offer overly positive feedback to an idealized therapist or may find it difficult to provide honest feedback (Bender, 2005; Meier, 2022). While assessment of alliance from the patient's perspective is an evidence-based practice (Fluckiger et al., 2018), additional ratings from therapists or external evaluators could have provided a broader picture of alliance development.

Fourth, based on the binary gender registration used in the clinic, the majority of the participants were female (87%). This skewed distribution is typical for most clinical BPD samples (Storebo et al., 2020), and limits the generalizability of the findings to male and gender-diverse individuals. In addition, as the vast majority of participants were young females, age and gender were not investigated as predictors in the LMM analyses. Accordingly, the findings are mainly applicable to young female patients.

## Conclusion

The study demonstrates overall satisfactory levels of therapeutic alliance in an MBT program for BPD that combines individual and group therapy. In the individual therapy, all aspects of the alliance were high, both initially and throughout treatment. In group therapy, the alliance was more complex. While patients reported positive bonding to the group in terms of group climate, group cohesion and alliance, there were uncertainties regarding tasks and goals in group therapy, particularly in the early phase. The study thus highlights challenges in ensuring that tasks and goals agreed upon in individual therapy are effectively implemented in the group therapy setting. Furthermore, the study points to somewhat strained relationships with other group members, both initially and over the course of treatment. Yet, as a “training ground” for relational functioning, these findings may indicate an appropriate level of interpersonal strain in MBT-G. The results further suggest that, in MBT, patients' initial level of RF may not affect their experience of therapeutic alliance. Future studies should investigate the relationships between RF, various aspects of alliance, and clinical outcome.

## Data Availability

The dataset used and analysed in the current study can be made available on special request to the corresponding author.
